# Crystal structure of the C-terminal domain of tubulin-binding cofactor C from *Leishmania major*

**DOI:** 10.1016/j.molbiopara.2015.05.003

**Published:** 2015-05

**Authors:** Keri L. Barrack, Paul K. Fyfe, Alex J. Finney, William N. Hunter

**Affiliations:** Division of Biological Chemistry & Drug Discovery, College of Life Sciences, University of Dundee, Dundee DD1 5EH, Scotland, UK

**Keywords:** ARL3, ADPribosylation factor-like protein 3, CARP, cyclase-associated proteins and X-linked retinitis pigmentosa 2 gene products, TEV, tobacco etch virus, TBC, tubulin-binding cofactor, LmTBCC, TBCC from *Leishmania major*, LmTBCC-C, C-terminal domain of the *L. major* protein, PEG 2000 MME, polyethylene glycol monomethyl ether, NMR, nuclear magnetic resonance spectroscopy, PDB, protein data bank, RP2, retinitis pigmentosa 2 protein, SAD, single-wavelength anomalous dispersion, SeMet, selenomethionine, β-helix, CARP domain, Crystal structure, Tubulin-binding protein

## Abstract

Tubulin-binding cofactor C stimulates GTPase activity and contributes to the release of the heterodimeric α/β-tubulin from a super-complex of tubulin monomers and two ancillary cofactors. We have determined the 2.2 Å resolution crystal structure of the C-terminal domain of tubulin-binding cofactor C from *Leishmania major* based on single wavelength anomalous dispersion measurements targeting a selenomethionine derivative. Although previously predicted to consist of two domains the structure is best described as a single domain dominated by a right-handed β-helix of five turns that form a triangular prism. One face of the prism is covered by the C-terminal residues leaving another face solvent exposed. Comparisons with an orthologous human GTPase activating protein match key residues involved in binding nucleotide and identify the face of the β-helix fold likely involved in interacting with the β-tubulin:GTP complex.

## Highlights

• The crystal structure of the C terminal fragment of tubulin binding cofactor C from *Leishmania major* is reported.
• The structure identifies a single domain dominated by a right handed β helix.
• Comparisons suggest key residues involved in stimulating the GTPase activity of β tubulin.

## Graphical abstract

The crystal structure of the C-terminal fragment of tubulin-binding cofactor C from *Leishmania major* has been determined. The structure identifies a single domain dominated by a right-handed β-helix and comparisons suggest key residues involved in stimulating the GTPase activity of β-tubulin.

**Figure:**
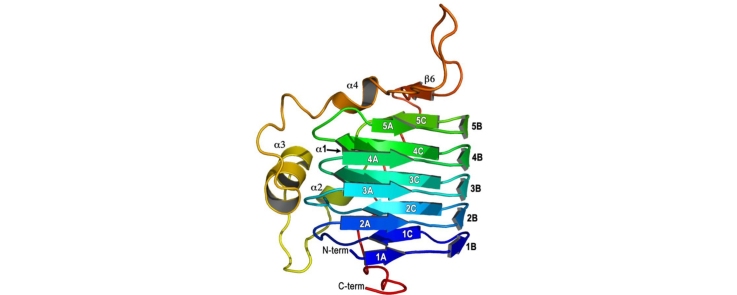

The cytoskeleton of trypanosomatids is highly enriched in microtubules. They form a network of subpellicular network beneath the cell membrane and maintain the overall parasite shape. Microtubules are abundant in the flagellum, an organelle important for pathogenicity as well as motility, for attaching the parasite to the salivary gland of the insect vector prior to the infection of a new host and also contributing to the evasion of the host immune system [1,2]. The folding and polymerization of α- and β-tubulin subunits is carefully regulated to ensure their correct tertiary structure and to prevent spontaneous aggregation or premature polymerization [3]. The assembly process involves distinct stages, each influenced by several chaperones or cofactors [4,5]. After a tubulin polypeptide is produced it is captured by prefoldin [6] then passed to the T-complex polypeptide 1 complex [7] where the folding is essentially completed. The tubulin-binding cofactors (TBC) are then involved in heterodimer assembly and polymerization [4,5,8]. The proteins involved are highly conserved across species and we are exploiting trypanosomatids as the model system to help dissect the contributions that they make to microtubule assembly [9–11].

There are five TBCs, termed A–E. TBCB and E bind α-tubulin whilst A and D interact with β-tubulin, to deliver each tubulin subunit into a super-complex comprising the α/β-tubulin heterodimer and cofactors D and E [8]. TBCC is involved in the final stage activation of GTP hydrolysis by β-tubulin, promoting release of the α/β-tubulin heterodimer from the super-complex protein assembly that can then proceed to polymerization [8,12,13]. This TBCC cofactor is a polypeptide of about 340 amino acids, located in the centrosome and predicted to form three distinct domains [14]. The N-terminal domain of the human protein has been characterized by nuclear magnetic resonance spectroscopy (NMR) and structures deposited in the Protein Data Bank (PDB) [14]. This N-terminal domain carries a flexible and unstructured N-terminus, that interacts with tubulin, and this leads into a bundle of three α-helices [14]. The fold is similar to that of TBCA [11]. NMR structures of a truncated C-terminal fragment of human TBCC, consisting of 179 amino acids are deposited in the PDB (code 2yuh, unpublished). This protein domain shares a sequence identity of approximately 20% with the corresponding domain of the trypanosomatid proteins.

Here we concentrate on TBCC from *L. major* (*Lm*TBCC, Uniprot code Q4Q1A3). We describe the crystallographic analysis of the C-terminal domain of *Lm*TBCC-C. Recombinant forms of the full-length protein from *Trypanosoma brucei* and *L. major* (335 amino acids, approximate mass 36.8 kDa) were prepared but proved recalcitrant to structural studies. In particular, speedy degradation of the polypeptides was noted. Limited proteolysis of the *L. major* protein, using trypsin, followed by mass spectrometry finger printing matched to the identification of a C-terminal fragment. A recombinant form of this domain, comprising residues 152–355 with a Leu223Met mutation was prepared (*Lm*TBCC-C) to allow the production of selenomethionine (SeMet) derivative protein. The strategy behind the mutation was to enhance the chances of obtaining a good anomalous dispersion signal by placement of selenium into the hydrophobic core of the protein fold at a position unlikely to influence the structure. Sequence comparisons (not shown) indicated that at positions corresponding to 223 a leucine, isoleucine (as in human TBCC PDB code 2yuh) or methionine is observed. This form was crystallized and the structure determined at 2.2 Å resolution by exploiting the anomalous dispersion X-ray scattering properties of selenium. Crystallographic details are presented in Table 1 and the coordinates and structure-factor data have been deposited in the PDB with accession code 5aj8.

Two polypeptides constitute the asymmetric unit and in each there are two segments, residues 155–157 and 326–355, which could not be modeled due to disorder*.* Non-crystallographic symmetry was not restrained during the refinement and it is noteworthy that the two molecules adopt a similar structure with an r.m.s.d for all atoms of just 0.77 Å between residues 158–325, reducing to just 0.18 Å when only the main chain atoms are considered. It is therefore only necessary to detail one molecule.

The N-terminal segment, residues 158–242, forms a right-handed parallel β-helix barrel consisting of five coils or layers. The helical barrel is shaped as an approximate triangular prism, with faces and strands labeled A, B, C (Fig. 1A). The sides are about 20 × 10 × 20 Å with height approaching 25 Å. Three short β-strands, A and C between four and six residues in length and B only 3 residues, together form each layer of the β-helix. This β-helix structure is classified as a CARP domain, identified in cyclase-associated proteins and X-linked retinitis pigmentosa 2 gene products (http://smart.embl-heidelberg.de/smart/do_annotation.pl?DOMAIN=CARP). The rest of the polypeptide is catalogued as the TBCC domain [14] but it forms an extended structure, not at all domain-like, mainly positioned over one side of the β-helix, the C-face. This section exits the β-helix, drops down (α1) then loops up (α2, α3) and over to place β6 as a cap of the β-helix (Fig. 1A). There are numerous interactions involving hydrogen bonds and van der Waals forces formed between residues in this extended segment and those on the β-helix, including a tyrosine “ladder” composed of Tyr188, Tyr207 and Tyr224, to stabilize the fold. These interactions involve main chain and side chain groups. The arrangement of the three tyrosine residues, together with some of the stabilizing hydrogen bonding interactions, is depicted in Fig. 1B. In addition a significant hydrophobic core formed by aromatic residues (Phe170, Trp226, Tyr244, Trp247, Trp267, Phe316) help to position the α-helical loop onto the C-face (not shown). The polypeptide crosses over the top of the β-helix then drops down and the C-terminal region is tucked under the cylindricαl structure interacting with the N-terminal residues*.* The overall result is that the so-called “TBCC domain” is actually an extended structure that forms a shield covering one side of the molecule leaving another to potentially interact with binding partners. The extended conformation and overall structure of residues 243–355 suggests this is not in itself a *bone fide* domain but rather contributes to an overall C-terminal domain.

A common feature of the right-handed β-helical fold is the existence of stacks of hydrophobic residues within the barrel [15]. *Lm*TBCC-C is no exception. A stack of four cysteine residues are positioned within the core of the helix in the middle of the βB strands 2 to 5, while nearby on βC strands 2 and 3 another two cysteine residues form a minimal stack, the sulfur atoms being 3.8–4.7 Å apart vertically within a stack separated by a distance of 5.2 Å horizontally between the two stacks. Aliphatic hydrophobic residues dominate this part of the structure and form the core of the β-helix.

The structure of the *Lm*TBCC-C domain matches to the human retinitis pigmentosa 2 (RP2) protein with an r.m.s.d. of 2.3 Å and a sequence identity of about 25% over 139 residues (Fig. 2A). An alignment of the amino acid sequences is presented in Fig. 2B. The β-helix structures are closely related and the majority of strictly conserved residues occur on the β-strands. This extends to a similar cysteine stacking formation to that discussed above [16]. A further stack of conserved aliphatic and hydrophobic residues is positioned in the middle of βA strands 2–5. The particular arrangement of externally oriented tyrosine residues in the middle of βC strands 1–4 also creates the same tyrosine “ladder” described. These conserved features suggest they are likely important for the correct folding and stability of the β-helix fold [17].

The RP2 protein is not involved in tubulin heterodimerization but like TBCC it does facilitate the GTPase activity of tubulin in the presence of TBCD [18,19]. RP2 forms a complex with Arf-like GTP-binding protein ARL3 (ADP ribosylation factor-like protein 3) and a crystal structure of the RP2:ARL3:GTP complex has been determined [16]. A comparison of the structures is instructive such that we are able to identify, with some confidence, the β-helix A face on *Lm*TBCC-C as the region for interaction with GTP and by implication β-tubulin. Strikingly, residues shown to be critical for RP2 activity are conserved. In particular *Lm*TBCC Arg214 and Glu234 correspond to RP2 Arg118 and Glu138. The arginine contributes directly to the catalytic activity of this type of GTPase, it interacts with the γ-phosphate, and is sometimes termed the arginine-finger [20]. Mutation of this arginine in TBCC has been shown to abolish GTPase stimulating activity [18]. Other key residues are conserved in and around the potential GTP:β-tubulin binding site on the β-helix A-face. In the RP2:ARL3:GTP complex, on one side of the catalytic arginine, a pair of glutamines (Gln115, Gln116) form hydrogen bonds to position the GTP ribose and α-phosphate moieties respectively. In *Lm*TBCC these correspond to His211 (not shown) and Gln212. Nearby, on the other side of the arginine, a basic patch is formed on both proteins, Arg120 in RP2, Lys216 in *Lm*TBCC. On the strand below Arg118/214 (A3), and directed onto the surface of the A-face, lie conserved serine (Ser99, Ser195) and phenylalanine (Phe101, Phe197) residues. These are positioned such that they would seem likely to be involved in protein–protein interactions.

Our structural data and the availability of *Lm*TBCC-C in a stable recombinant form can now support and inform further studies to elucidate the precise contributions that these residues make to the final stage processing of the α/β-tubulin heterodimer.

## Figures and Tables

**Fig. 1:**
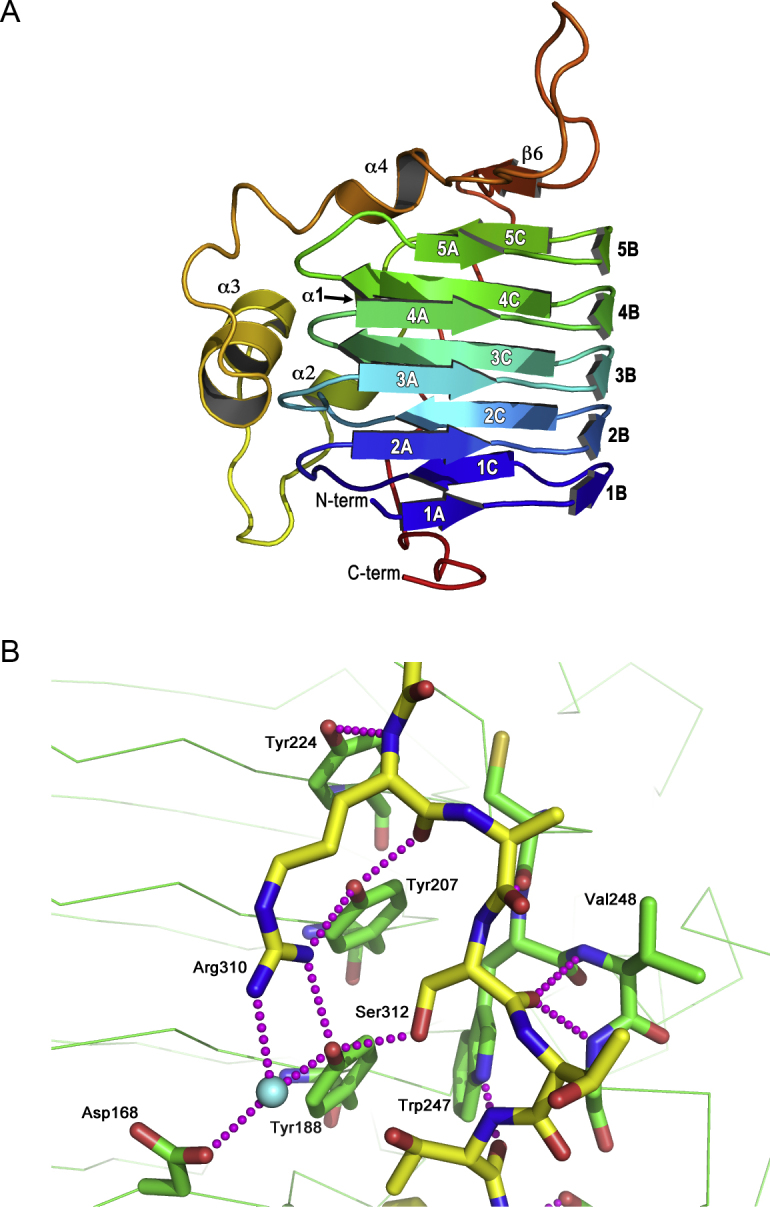
The structure of *Lm*TBCC-C. (A) A ribbon diagram of the fold, colored blue to red from N-terminus to C-terminus. Helix α1, is obscured behind the β-helix structure in this orientation. (B) The tyrosine ladder and a nearby tryptophan (green C atoms) and stabilizing hydrogen bonding interactions (purple dashed lines) formed with the extended C-terminal residues (yellow C atoms). A water molecule is depicted as a cyan sphere, N and O atoms are colored blue and red respectively (For interpretation of the references to color in this figure legend, the reader is referred to the web version of this article.).

**Fig. 2:**
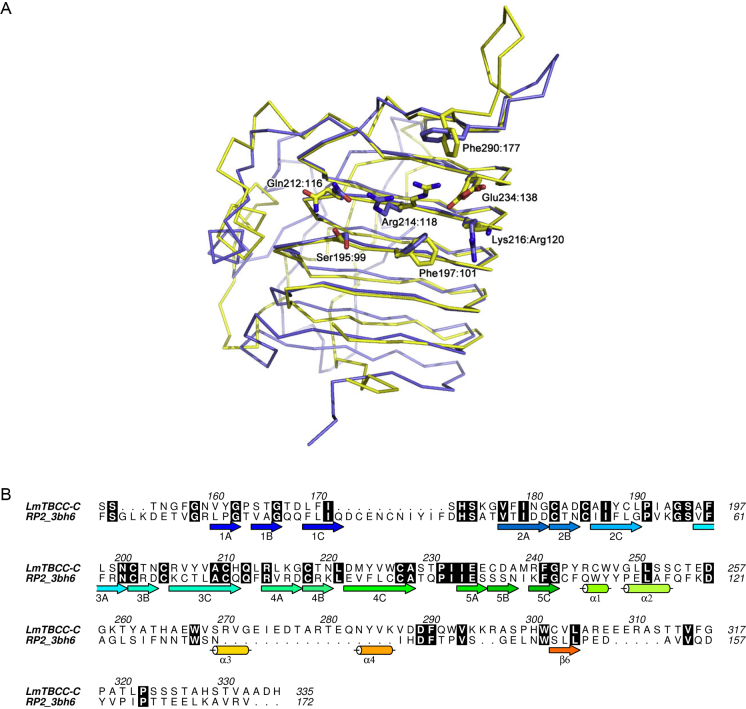
Comparison of *Lm*TBCC-C with human RP2. (A) RP2 (blue Cα, PDB 3βh6 [19]) superimposed on *Lm*TBCC-C (yellow Cα). The two proteins share 24% sequence identity. DALI [31] was used to inform comparison with structures in the PDB. The second domain from RP2 (residues 224–350) for which there is no similarity to *Lm*TBCC-C has been removed for clarity. Several residues implicated in GTP binding in the RP2-ARL structure are highlighted where they remain conserved or conservatively altered in *Lm*TBCC-C. These are labeled with the *Lm*TBCC residue type and number, then the RP2-ARL residue type if different, and number. Residues positioned to make hydrogen bond interactions with the nucleotide from RP2 and their corresponding residues in *Lm*TBCC-C are shown as sticks. (B) Sequence alignment of *Lm*TBCC-C with part of human RP2 (Uniprot: O75695). The secondary structure of *Lm*TBCC-C is shown and strictly conserved residues encased in black. Figure made with ALINE [32].

**Table 1:** Crystallographic details of *Lm*TBCC-C.

Data collection	
Wavelength (Å)	0.97907
Space group	P2_1_

Cell dimensions	
a, b, c (Å)a, b, g (°)	37.6 93.2 48.390.0 108.4 90.0
Resolution (Å)	46.6–2.2 (2.27–2.20)^a^
R_merge_/<I/sI>	10.2 (23.9)/17.5 (6.8)
CC _½_^b^	0.998 (0.967)
Completeness (%)/multiplicity	98.3 (86.8)/11.9 (7.1)
Anomalous completeness	97.7 (82.5)
Anomalous multiplicity	6.0 (3.4)
Wilson B(Å^2^)	14.6

Refinement	
No. reflections (total/Rfree)	15026 (792)
Rwork/Rfree	15.8/19.7
No. atoms protein/water	2697/300

B-factors(Å^2^)	
Protein (chainA/chain B)	19.8/20.1
Water	28.4

r.m.s. Deviations	
Bond lengths (Å)/angles (°)	0.010/1.326

Ramachandran distribution (%)^c^	
Favored/outliers	98.2/0

The gene fragment encoding residues 152–355 of *Lm*TBC-C from *Leishmania major* strain Friedlin identified in GeneDB (LmjF.36.3160, [21]) was amplified from genomic DNA using PCR. To permit the use of selenomethionine for phase determination a single mutation, Leu223Met was introduced (Quikchange mutagenesis, Stratagene). The gene was cloned into a modified pET15b plasmid to encode an N-terminal His-tag followed by a tobacco etch virus (TEV) protease cleavage site. The resulting vector was transformed into *Escherichia coli* B834 (DE3), and cells grown in Selenomethionine Medium (Molecular Dimensions, UK), expression induced with 1 mM IPTG at an OD_600_ 0.6 and growth continued at room temperature for 16 hours. Cells were harvested by centrifugation and resuspended in 50 mM Tris–HCl pH 7.5, 250 mM NaBr, 20 mM imidazole before storage at −20 **°**C. Thawed cells were lysed using French Press at 16 kpsi and lysate was clarified by centrifugation at 37,500 × *g* for 30 min at 4 °C. Soluble supernatant was filtered (0.2 μm) and loaded onto a 5 mL HisTrap HP column (GE Healthcare) pre-equilibrated with 50 mM Tris–HCl, 250 mM NaBr pH 7.5 for an initial affinity chromatography capture step. Elution of *Lm*TBCC-C was performed by applying an imidazole gradient with the target protein eluting at approximately 140 mM. The product was treated with TEV protease at 30 °C for 2 h. Dialysis at room temperature, to remove excess imidazole, was followed by reverse affinity chromatography prior to a final purification step with size exclusion chromatography using a calibrated Superdex 200 26/60 gel filtration column and the equilibration buffer. The protein eluted with an estimated mass of 20 kDa, which corresponds to that expected for a monomeric sample (20.4 kDa). The sample was pooled, buffer exchanged into 10 mM Tris–HCl, 100 mM NaBr pH 7.5 and concentrated using a centrifugal concentrator (10 kDa cutoff, Sartorius) prior to crystallization. The protein concentration was determined by measurement of absorbance at 280 nm and an estimated extinction coefficient 38,680 M^−1^ cm^−1^[22]. Poor quality crystals were produced at 18 °C by the hanging drop vapor diffusion method using 0.75 μL of protein solution at a concentration of 7 mg mL^−1^, mixed with 0.75 μL of reservoir containing 100 mM MES (4-morpholineethanesulfonic acid) pH 6.5, 25–30 % PEG 2000 MME (polyethylene glycol monomethyl ether). A crystal was placed into an Eppendorf tube with 100 μL of reservoir and a small nylon ball was added before vortexing for 30 seconds to create a micro-crystal suspension. Fresh conditions were prepared with 100 mM MES pH 6.7, 22% PEG 2000 MME in the reservoir, and protein solution as before but at a reduced concentration of 3 mg mL^−1^. A cryo-loop was used to streak the micro-seed suspension into the conditions and the plates stored at 18 °C. Well formed needles (40 × 40 × 500 mm) appeared in several days. Single-wavelength anomalous dispersion (SAD) data were measured from a single crystal at −170 °C on beam line I24 of the Diamond Light Source with a Pilatus 6 M detector. A helical data collection protocol to minimize radiation damage was used. Data were indexed and integrated using XDS [23] and scaled using AIMLESS [24]. The structure was solved via SAD-phasing using Phenix AutoSolv [25]. Two molecules of *Lm*TBCC-C constitute the asymmetric unit and each contains two SeMet residues. These four Se positions were identified and provided an initial figure-of-merit 0.44. The density modification step yielded an improved figure-of-merit of 0.69 and was followed by automated model building to produce a partial model consisting of 311 residues giving an R/R_free_ of 24.2%/29.1% and a map-model correlation coefficient of 0.79. The model was then completed with the graphics software COOT [26]. Refinement was performed in REFMAC5 [27] utilizing Translation/Libration/Screw refinement [28], and alternated with rounds of electron and difference density map inspection and model manipulation together with ligand incorporation using COOT, and the incorporation of waters and alternate conformer side chains. Non-crystallographic symmetry restraints were not employed. MOLPROBITY [29] was used to investigate model geometry in combination with the validation tools provided in COOT.

a Values in parenthesis are for the highest resolution shell.

b Pearson correlation coefficient [30].

c Calculated using the Molprobity server (http://molprobity.biochem.duke.edu).
